# Mental health status of North Korean refugees in South Korea and risk and protective factors: a 10-year review of the literature

**DOI:** 10.1080/20008198.2017.1369833

**Published:** 2017-09-04

**Authors:** Yeeun Lee, Minji Lee, Subin Park

**Affiliations:** ^a^ Department of Research Planning, Mental Health Research Institute, National Center for Mental Health, Seoul, South Korea

**Keywords:** North Korean refugee, mental health, post-traumatic stress disorder, depression, anxiety

## Abstract

**Background**: North Korean refugees (NKRs) are often exposed to traumatic events in North Korea and during their defection. Furthermore, they face sociocultural barriers in adapting to the new society to which they have defected.

**Objective**: To integrate previous findings on this mentally vulnerable population, we systematically reviewed articles on the mental health of NKRs in South Korea.

**Method**: We searched for empirical studies conducted in the last 10 years in six online databases (international journals: Embase, PubMed, Scopus, Web of Science; Korean journals: DBPIA, KMbase) through June 2017. Only quantitative studies using new empirical data on the mental health of NKRs were included. We summarized the 56 studies ultimately selected in terms of NKRs’ mental health status and three domains of associated factors: pre- and post-settlement factors and personal factors.

**Results**: NKRs had a high prevalence and severity of psychiatric symptoms, particularly post-traumatic stress disorder and depression. We identified nine risk factors consistently found in previous studies, including traumatic experience, longer stay periods in third country, forced repatriation, acculturative stress, low income, older age, poor physical health, and female and male sex, as well as four protective factors, including educational level in North Korea, social support, family relationship quality, and resilience.

**Conclusions**: We suggest that future studies focus on the causal interactions between different risk and protective factors and mental health outcomes among NKRs from a longitudinal perspective. Furthermore, comprehensive policies for NKRs’ psychological adaptation are needed, particularly the development of evidence-based mental health interventions.

## Abstract (es)

**Planteamiento:** Los refugiados de Corea del Norte (RCN) suelen estar expuestos a eventos traumáticos en Corea del Norte y durante su deserción. Además, se enfrentan a barreras socioculturales a la hora de adaptarse a la nueva sociedad a la que han desertado.

**Objetivo:** Para integrar los hallazgos previos sobre esta población mentalmente vulnerable, revisamos sistemáticamente artículos sobre la salud mental de los RCN en Corea del Sur.

**Método:** Buscamos estudios empíricos realizados en los últimos 10 años en seis bases de datos en internet (revistas internacionales: Embase, PubMed, Scopus, *Web of Science*, revistas coreanas: DBPIA y KMbase) hasta junio de 2017. Solo se incluyeron estudios cuantitativos que usaban nuevos datos empíricos sobre la salud mental de los RCN. Se resumieron los 56 estudios seleccionados en última instancia en términos de estado de salud mental de los RCN y tres dominios de factores asociados: factores pre y post-establecimiento y factores personales.

**Resultados:** Los RCN presentaron una alta prevalencia y gravedad de síntomas psiquiátricos, particularmente trastorno de estrés postraumático y depresión. Se identificaron nueve factores de riesgo encontrados consistentemente en estudios previos, los cuales incluyen experiencias traumáticas, períodos de permanencia más prolongados en el tercer país, repatriación forzada, estrés por aculturación, bajos ingresos, edad avanzada, mala salud física, y sexo femenino y masculino, así como cuatro factores de protección, que incluyen nivel educativo en Corea del Norte, apoyo social, calidad de la relación en la familia y la capacidad de recuperación o resiliencia.

**Conclusiones:** Sugerimos que en el futuro, los estudios se centren en las interacciones causales entre los diferentes factores de riesgo y de protección y los resultados de salud mental entre los RCN desde una perspectiva longitudinal. Además, se necesitan políticas globales para la adaptación psicológica de los RCN, en particular el desarrollo de intervenciones de salud mental basadas en la evidencia.

## Abstract (zh)

**背景：**朝鲜难民（NKRs）在朝鲜以及脱北过程中经常经受创伤事件。因此，他们在适应和朝鲜不同的新社会时会面临社会文化壁垒。

**目标：**为了整合过去关于这个精神脆弱群体的研究发现，我们系统地回顾了关于NKRs在韩国的精神健康的文章。

**方法：**我们在6个在线数据库里（国际期刊：Embase, PubMed, Scopus, Web of Science；韩国期刊：DBPIA and KMbase）搜索了截止2017年7月过去10年间的实证研究。只包括了关于NKRs精神健康的使用新的实证数据的量化研究。我们总结了最终选择的56个研究中关于NKRs的净胜健康状况和三个相关的因素：安置前、安置后因素和个人因素。

**结果：**NKRs的精神病性症状表现高患病率和高严重性，尤其是创伤后应激障碍和抑郁。我们识别了9个在前人研究中一致被报告的风险因子，包括创伤经历、在第三国更长的停留时间、被强迫遣返、文化适应压力、低收入、年老、身体健康状况差以及男女性别。同时还识别了4个保护性因子，包括：在朝鲜的教育水平、社会支持、家庭关系质量和韧性。

**结论：**我们建议，未来的研究可集中在从追踪视角对不同风险和保护因子对NKRs的精神健康结果的因果相互关系进行讨论。进一步地，需要制定NKRs心理适应的综合政策，尤其是开发循证的精神健康干预方案。

## 1. Introduction

The number of North Korean refugees (NKRs) who have settled in South Korea exceeded 30,000 in 2016 (Ministry of Unification, 2017). The population consists of individuals across a wide age range, particularly young and middle-aged adults, with an approximate women’s ratio of 0.8. According to the census data, the most frequent drives for their defection include food shortage, economic difficulty, and political repression or threat in North Korea, and family accompaniment in South Korea (Korea Hana Foundation, 2017). In most cases, NKRs take an escape route through a third country to reach South Korea, mainly Southeast Asian countries or China (Haggard & Marcus, 2006), and about 70% of NKRs reported to have stayed in the third country, half of whom stayed more than five years (Korea Hana Foundation, 2017).

Similar to other refugee populations (Fazel, Wheeler, & Danesh, 2005; Hollifield, Warner, & Lian et al., 2002), NKRs are often exposed to traumatic events while in North Korea and during their escape (Jeon, Yu, Cho, & Eom, 2008). Even after a successful escape, they tend to experience difficulties in adapting to the unfamiliar culture of the country in which they settle (Jeon, Min, Lee, & Lee, 1997). For instance, a quarter of NKRs who settled in South Korea reported experiencing discrimination, mostly due to cultural differences in language use, lifestyle, and attitudes (Korea Hana Foundation, 2017). Therefore, NKRs might be a mentally vulnerable group. Although an increasing number of studies have examined the mental health of NKRs, to our knowledge, there have been no systematic reviews integrating the existing knowledge of this topic. Thus, in an effort to produce such an integrative understanding, we reviewed prior studies on the mental health status of NKRs and the associated risk and protective factors. We could not conduct a meta-analysis because of the methodological and clinical heterogeneity of the studies. Thus, we instead systematically searched the studies using particular selection criteria, summarized the prevalence of psychiatric symptoms among NKRs, and identified consistent risk and protective factors. This study first categorized a wide array of risk and protective factors into environmental and personal factors, and further divided the environmental factors according to the different phases of the refugee experience into pre- and post-settlement factors, as has been done in other review studies of refugee populations (Lustig et al., 2004; Porter & Haslam, 2005).

## 2. Methods

### 2.1. Inclusion and exclusion criteria

We included quantitative studies using new empirical data on the mental health of NKRs in South Korea. We selected studies published in the last 10 years, with a minimum sample size of 25. As such, qualitative studies, reviews, and unpublished findings were excluded from our review. Furthermore, we excluded studies on physical health, efficacy of intervention, general adaptation, and the development and validation of psychological assessments. We searched only for studies written in English or Korean.

### 2.2. Search strategy

**Figure 1.**
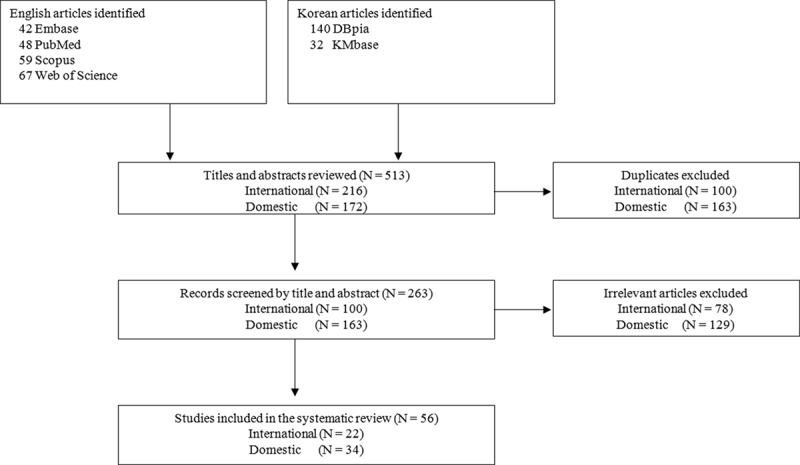
Search strategy and article selection process.

We searched for empirical studies conducted in the last 10 years in six online databases (international journals: Embase, PubMed, Scopus, Web of Science; Korean journals: DBpia, KMbase) that are published up to June 2017. As search terms, we used the combinations of keywords relating to mental health (psychiatr*, psycholog*, mental, anxiety, depression, trauma, traumatic, psychosocial, wellbeing, recovery, resilience, adjustment, adaptation, emotion, and behavior) and North Korean refugees (North Korean defector and North Korean refugee) in each database language. The search strategy was adjusted for each database. With these search terms, 513 studies were initially identified. After excluding duplicate or irrelevant studies by reviewing titles and abstracts, a total of 56 studies were ultimately included (Figure 1). The included studies are summarized in Table 1.

### 2.3. Data synthesis and analysis

The prevalence of psychiatric symptoms reported in the included studies was summarized by symptoms, including anxiety, depression, PTSD, insomnia, and overall psychological health. We then categorized the associated risk and protective factors into three domains: pre-settlement factors (educational background, traumatic events, immigration process), post-settlement factors (time since resettlement, acculturative stress, social support, family relationships, socioeconomic status), and personal factors (individual characteristics, sex, age, condition). After that, we identified consistent risk and protective factors using an approach employed in previous systematic reviews (Cannon, Jones, & Murray, 2002; Fazel, Reed, Panter-Brick, & Stein, 2012): only those factors that were consistently found to be associated with mental health outcomes in the same direction in three or more studies were included. When at least one study reported the opposite association between the same factor and mental health outcome, the factor was excluded.

## 3. Results

### 3.1. Epidemiology of mental health problems

The prevalence of mental health problems among NKRs is summarized in Table 2. Firstly, for emotional disorders, 33–51% of NKRs were classified as having depressive symptoms in eight studies (Ahn et al., 2012; Choi, Min, Cho, Joung, & Park, 2011; Jeon et al., 2009; Kim et al., 2011; Kim, Kim, Kim, Cho, & Lee, 2011; Lee et al., 2007; Y. Lee, J. Jun, Y. Lee et al., 2016; Nam, Kim, DeVylder, & Song, 2016); and 43–54% of NKRs were classified as having anxiety symptoms in two studies (Ahn et al., 2012; Choi et al., 2011). When using a single scale for both depression and anxiety symptoms, 10–48% of NKRs were classified as having a clinical level of depression and anxiety symptoms in four studies (Cho & Kim, 2010; Y. Kim, 2013; Kim, Jeon, & Cho, 2010; Shin, Lee, & Park, 2016). Furthermore, there were four case-control studies that compared depression and anxiety symptoms between NKRs and South Koreans (Kim, Choi, & Chae, 2008; Y. Lee, J. Jun, Y. Lee et al., 2016; Lee et al., 2016; Shin et al., 2016), all of which consistently reported higher prevalence or average level for NKRs. These findings suggest that NKRs may be more susceptible to emotional disturbances including depressive and anxiety symptoms.

**Table 1.** Included studies.

Author (year)	Study population	*N*	Mean age (range)	Outcomes (assessment tools)	Comparison group
Ahn et al. (2012)	Adult NKRs	56	39.3	Depression (BDI), Anxiety (BAI)	No
Baek et al. (2007)	Adolescent and young adult NKRs	200	19.3 (11–29)	Emotional and behavioural problems (K-YSR)	No
Chae and Kim (2014)	Adult NKRs	82	34.4 (18–60)	Interaction Anxiousness (IAS)	Adult South Koreans
Chang and Son (2014)	Adult NKRs	81	37.9	PTSD (PDS, CPTSD-I), Psychiatric symptoms (SCL-90-R)	Adult South Koreans
Cho and Kim (2010)	Female adult NKRs	401	36.0 (21–59)	PTSD (SCID), Anxiety/Depression (HSCL-25)	No
Cho et al. (2011)	Adolescent NKRs	195	18.0 (10–23)	Internalized and externalized problems, PTSD (co-author’s measurement)	No
Cho et al. (2009)	Adult NKRs	106	35.3	Depression (BDI), Anxiety (HSCL-25)	No
B. Choi and Kim (2011)	Female adult NKRs	1936	34.7 (18–69)	Psychiatric symptoms (MMPI-2, SCL-90-R)	No
S. Choi et al. (2011)	Adolescent and young adult NKRs	108	19.4 (12–29)	Anxiety/Depression (HADS)	No
Y. Choi et al. (2009)	Male adult NKRs	227	35	Personality and Psychopathology (K-PAI)	No
Y. Choi et al. (2017)	Adult NKRs	211	38.4	Depression (CES-D), Anxiety (STAI), PTSD (IES-R), Somatization (SCL-90-R)	No
Emery et al. (2015)	Adolescent NKRs, China born children of NKRs	82	18.0 (15–25)	Depression (CES-D)	No
B. Jeon et al. (2009)	Adult NKRs	367	40.4	Depression (CES-D)	No
W. Jeon et al. (2013)*	Adult NKRs	106	35.3	PTSD (SCID), Depression (BDI), Anxiety/Depression (HSCL-25)	No
W. Jeon et al. (2008)	Adult NKRs	62	34.3 (19–55)	Personality and Psychopathology (K-PAI)	No
Jun et al. (2015)	Adult NKRs	201	38.8	Depression (CES-D), Anxiety (STAI), Somatization (SCL-90-R), PTSD (IES-R).	No
J. Jung et al. (2013)	Adult NKRs	198	39.3	Psycho-social adaptation (author’s measurement)	No
Y. Jung and Choi (2017)	Female adult NKRs	416	35.3	Psychological symptoms (BPSI-NKR)	No
H. Kim (2010)	Female adult NKRs	283	35.4	Psychiatric symptoms (SCL-90-R)	No
H. Kim (2012a)	Female adult NKRs	219	37.5	Depression (CES-D)	Female South Koreans
H. Kim (2012b)	Adult NKRs	531	34.6	Complex PTSD (CPTSD-I), PTSD (PDS), Depression (CES-D)	No
H. Kim (2013)	Adolescent NKRs	300	N/R (15–24)	Depression (BDI), Anxiety/Depression (HSCL-25), PTSD (DSM-IV), PTG (SRG)	No
H. Kim et al. (2011)	Adult NKRs	144	40.4	Psychiatric symptoms (SCL-90-R), Depression (CES-D)	No
H. Kim and Oh (2010)	Female adult NKRs	1,465	34.8	Psychological symptoms (MMPI-2)	No
H. Kim and Shin (2010)	Adult NKRs	484	N/R (above 20)	Psychological symptoms (BPSI-NKR)	No
H. Kim and Shin (2015)	Adolescent NKRs	202	N/R (14–19)	Psychological problems (PSI-NKR-A)	Adolescent South Koreans
J. Kim et al. (2008)	Adult NKRs	40	31.1	Depression (CES-D)	Adult South Koreans
J. Kim et al. (2017)	Female adult NKRs	140	N/R (above 20)	Depression (CES-D), PTSD (DSM-IV), Alcohol problem (AUDIT), Suicide ideation (SSI)	No
L. Kim et al. (2014)	Adolescent NKRs	156	17.7 (13–21)	Depression, Anxiety (K-YSR)	No
S. Kim et al. (2011)	Adult NKRs	144	40.4	Depression (CES-D)	Adult South Koreans
S. Kim et al. (2013)	Female adult NKRs	2,163	34.7 (18–69)	Psychological symptoms (MMPI-2)	No
S. Kim et al. (2016)	Male adult NKRs	272	35.9	Nicotine dependence (KTSND)	No
Y. Kim (2013)	Adolescent NKRs	200	18.2 (13–21)	Anxiety/Depression (HSCL-25), PTSD (PDS)	No
Y. Kim (2016)	Adolescent NKRs	144	18.2 (13–21)	PTSD (PDS), Complex PTSD (DESNO), Anxiety/Depression (HSCL-25)	No
Y. Kim et al. (2015)	Adolescent NKRs	144	18.2 (13–21)	Anxiety/Depression (HSCL-25), PTSD (UCLA Posttraumatic Stress Disorder Index)	No
Y. Kim et al. (2010)	Adult NKRs	500	N/R (20–62)	Anxiety/Depression (HSCL-25), PTSD (SCID)	No
I. Lee et al. (2011)	Children NKRs	103	9.9 (7–14)	Psychological problems, Emotional and behavioural problems (K-YSR)	No
K. Lee (2011)	Adolescent NKRs	172	N/R	Anxiety/Depression (HSCL-25)	No
K. Lee et al. (2007)	Adolescent and adult NKRs	87	N/R	Depression (CES-D)	No
M. Lee et al. (2016)	Female adult NKRs	87	39.2	Depression (CES-D)	No
Y. Lee, et al. (2016a)	Adult NKRs	177	38.2	Insomnia (questionnaire based on ICD-10), Depression (CES-D), PTSD (IES-R)	Adult South Koreans
Y. Lee, et al. (2016b)	Adult NKRs	45	35.4	Depression (BDI), Anxiety (BAI), PTSD (IES-R), Dissociation (DES-II)	Adult South Koreans
Y. Lee et al. (2012)	Adolescent NKRs	102	16.4	Emotional and behavioural problems (K-CBCL)	Adolescent South Koreans
J. Lim et al. (2010)	Adult NKRs	202	35.6	Psychological symptoms (PSI-NKR-A)	No
Y. Lim and Han (2016)	Adult NKRs	445	40.3	PTSD (a scale developed for NKRs)	No
Nam et al. (2016)	Adult NKRs	304	41.0	Depression (CES-D)	No
J. Park et al. (2015)	Adult NKRs	199	38.6 (19–74)	PTSD (IES-R), Depression (CES-D)	No
Y. Park and Yoon (2007)	Adolescent NKRs	197	19.2 (14–24)	Emotional and behavioural problems (K-YSR)	No
G. Shin and Lee (2015)	Female adult NKRs	97	36.5	PTSD (SCID), Psychiatric symptoms (SCL-47)	No
H. Shin and Kim (2015)	Adolescent NKRs	380	18.4	PTSD (PSI-NKR-A)	No
H. Shin et al. (2016)	Adult NKRs	593	40.7	Anxiety/Depression (a single question adopted from the KNHANES)	Adult South Koreans
Son et al. (2010)	Adolescent NKRs	146	18.3 (14–22)	Psychiatric symptoms (SCL-90-R)	No
B. Song et al. (2011)	Adult NKRs	32	50.2 (20–76)	Depression (BDI), PTSD (MMPI-II)	No
D. Song et al. (2016)	Adult NKRs	200	47.7	Depression (PHQ-9)	No
Um et al. (2015)	Adult NKRs	216	41.0	Depression (CES-D)	No
Yoo et al. (2017)	Female NKRs	242	40.5	Somatization (SCL-90-R)	No

*indicates prospective study

**Mean ages have been rounded off to the nearest tenth

BDI = Beck Depression Inventory, BAI = Beck Anxiety Inventory, K-YSR = Korean Youth Self-Report, IAS = Interaction Anxiousness Scale, PTSD = Post-Traumatic Stress Disorder, PDS = Posttraumatic Diagnostic Scale, CPTSD-I = Complex PTSD scale-I, SCL-90-R = Symptom Checklist-90-Revision, SCID = Structured Clinical Interview, HSCL-25 = Hopkins Symptoms Checklist-25, MMPI-2 = Minnesota Multiphasic Personality Inventory-2, HADS = Hospital Anxiety and Depression Scale, K-PAI = Korean version of Personality Assessment Inventory, CES-D = Center for Epidemiological Studies-Depression Scale, STAI = State-Trait Anxiety Inventory, IES-R = Impact of Event Scale-Revised, BPSI-NKR = Brief Psychological State Inventory for North Korean Refugees, DSM-IV = Diagnostic and Statistical Manual of Mental Disorders-IV, PTG = Post-Traumatic Growth, SRG = Stress-Related Growth, PSI-NKR-A = Psychological State Inventory for North Korean Adolescent Refugees, AUDIT = the Alcohol Use Disorders Identification Test, SIS = Suicide Ideation Scale, KTSND = Kano test for social nicotine dependence, DESNO = Disorders of Extreme Stress Not Otherwise Specified, DES-II = Dissociative Experiences Scale II, K-CBCL = Korean version of Child Behaviour Check List, KNHANES = Korea National Health and Nutrition Examination Survey, PHQ-9 = Patient Health Questionnaire-9

**Table 2.** The prevalence of mental health problems.

Psychiatric symptoms	Population	*N*	Assessment tool	Definition	Prevalence	Author (year)
Anxiety	Adult NKRs	56	BAI	Cut-off score: 22	43%	Ahn et al. (2012)
Anxiety	Adolescent NKRs	108	HADS	Cut-off score: 8	54%	S. Choi et al. (2011)
Depression	Adult NKRs	56	BDI	Cut-off score: 16	39%	Ahn et al. (2012)
Depression	Adult NKRs	367	CES-D	Cut-off score: 21	33%	B. Jeon et al. (2009)
Depression	Adult NKRs	144	CES-D	Cut-off score: 21	49%	H. Kim et al. (2011)
Depression	Adult NKRs	144	CES-D	Cut-off score: 21	51%	S. Kim et al. (2011)
Depression	Adolescent and Adult NKRs	87	CES-D	Cut-off score: 16	47%	K. Lee et al. (2007)
Depression	Adult NKRs	177	CES-D	Cut-off score: 21	46%	Y. Lee et al. (2016a)
Depression	Adult NKRs	304	CES-D	Cut-off score: 24	44%	Nam et al. (2016)
Depression	Adolescent NKRs	108	HADS	Cut-off score: 8	36%	S. Choi et al. (2011)
Depression and anxiety	Female adult NKRs	401	HSCL-25	Cut-off score: 1.75	10%	Cho and Kim (2010)
Depression and anxiety	Adolescent NKRs	200	HSCL-25	Cut-off score: 1.75	31%	Y. Kim (2013)
Depression and anxiety	Adult NKRs	500	HSCL-25	Cut-off score: 1.75	48%	Y. Kim et al. (2010)
Depression and anxiety	Adult NKRs	593	Self-report questions	Not reported	29%	H. Shin et al. (2016)
PTSD	Adult NKRs	81	PDS	Cut-off score: 15	52%	Chang and Son (2014)
PTSD	Female adult NKRs	401	SCID	Cut-off score: 19	4%	Cho and Kim (2010)
PTSD	Adult NKRs	432	PDS	Not reported	15%	H. Kim (2012b)
PTSD	Adolescent NKRs	200	PDS	Cut-off score: 21	13%	Y. Kim (2013)
PTSD	Adult NKRs	500	SCID	DSM-IV criteria	5%	Y. Kim et al. (2010)
PTSD	Adult NKRs	177	IES-R	Cut-off score: 25	40%	Y. Lee et al. (2016a)
Insomnia	Adult NKRs	177	Self-report questions	ICD-10 criteria	38%	Y. Lee et al. (2016a)

*The prevalence has been rounded up to the nearest whole number

Regarding PTSD, one of the most well-researched mental problems in refugee populations (Fazel et al., 2005), six studies reported the prevalence of PTSD using clinical criteria (Chang & Son, 2014; Cho & Kim, 2010; Kim, 2012b, Y. Kim, 2013; Kim et al., 2010; Lee et al., 2016). There were huge variations in reported prevalence, from 4% to 52%. The variations among these studies might be due to using different diagnostic methods and criteria, age ranges of participants, and sampling methods, which can partially explain the heterogeneity of results seen in refugee studies (Fazel et al., 2005). Nevertheless, when comparing with South Koreans, NKRs had a higher average level of PTSD symptoms in a case-control study (Chang & Son, 2014).

Beyond the aforementioned psychiatric problems, the prevalence of insomnia in NKRs (38%) was more than four times higher than in South Koreans (9%) in a study (Lee et al., 2016). Additionally, when comparing general psychological adaptation, adolescent NKRs reported higher levels of psychological problems, including post-traumatic stress, somatic symptoms, and attention and thought problems, and lower social functioning than their South Korean peers (Kim & Shin, 2015; Lee, Shin, & Lim, 2012). In general, both adult and adolescent NKRs are suggested to experience more mental difficulties in comparison with South Koreans.

### 3.2. Risk and protective factors

#### 3.2.1. Pre-settlement factors

##### 3.2.1.1. Educational background

The results regarding mental health effects of educational background (i.e. years of education or educational level) were mixed. Higher educational level in North Korea was inversely associated with emotional and behavioural problems of young and adult NKRs in several studies (Cho, Kim, & Kim, 2011; Choi, Lee, & Kim, 2009; Kim, 2010; Kim et al., 2010; Song, Shin, Lee, Kim, & Jun, 2016), but not in others (Chang & Son, 2014; Cho, Kim, & You, 2009; Kim, Kim, & Lee, 2013; Nam et al., 2016). In one 3-year follow-up study, NKRs who received higher education in North Korea exhibited less depressive symptoms at the beginning of the study; however, this association disappeared after three years (Cho, Jeun, Yu, & Um, 2005). Given the positive effect of educational background in enhancing resilience against external stressors (Bonanno, Galea, Bucciarelli, & Vlahov, 2007; Campbell-Sills, Forde, & Stein, 2009), education in North Korea might have a protective effect for adaptation difficulties, particularly in the earlier stage of resettlement.

##### 3.2.1.2. Traumatic events

A high proportion of young and adult NKRs are often exposed to multiple traumatic events while in North Korea or during their flight. Specifically, 49.3–81.4% of adult NKRs reported having directly experienced or witnessed at least one type of life-threatening events (Kim, 2012b; Nam et al., 2016), including starvation, public execution, and being sent to a correction facility (Jeon et al., 2008). Adolescent NKRs are not an exception: around 71% of adolescents NKRs reported having undergone at least one traumatic incident in one report (Kim, 2013), and the average number of traumatic incidents was 2.5 in another report (Kim, 2016). Moreover, female NKRs are under the additional risk of sexual violence: almost one-fourth of female NKRs reported lifetime sexual victimization, such as sexual harassment, rape, and sex labour, particularly in North Korea and third countries (Kim, Kim, Choi, & Nam, 2017).

Consistent with a meta-analysis of refugee populations that identified traumatic events as the strongest mental health risk factor (Steel et al., 2009), the number and severity of these traumatic experiences were all predictive of a wide range psychiatric disorders among NKRs in most studies (Baek, Kil, Yoon, & Lee, 2007; Cho & Kim, 2010; Cho et al., 2011; Choi et al., 2017; Emery, Lee, & Kang, 2015; Jeon, Eom, & Min, 2013; Kim, 2012b, Y. Kim 2013; Kim, Cho, & Kim, 2015; Lee et al., 2016; Lim & Han, 2016; Park et al., 2015). However, in a few studies (Cho et al., 2009; Jeon et al., 2013; Kim, 2016), traumatic experiences were not related to conditions other than PTSD, such as anxiety and depressive symptoms. A path analysis further noted the mediating role of PTSD symptoms in the link between traumatic experience and co-morbid psychiatric symptoms (Kim, 2016), suggesting a relatively indirect effect of traumatic events on co-morbid conditions other than PTSD. Furthermore, the mental health consequences of traumatic experience may differ according to the sub-types of incidents. When sub-typing traumatic events, repatriation, disease-related events, and interpersonal but not accidental events were predictive of NKRs’ PTSD and depression symptoms (Kim, 2012b; 2016).

##### 3.2.1.3. Immigration process

During the immigration process to South Korea, most NKRs pass through third countries such as China or Russia (Choi et al., 2009), and the average duration of stay in these third countries is approximately 2–5 years (Choi et al., 2009; Kim et al., 2011). So far, the mental health effects of longer stay periods in third countries have shown variable results. While some studies noted no significant association between length of stay and NKRs’ psychiatric symptoms (Ahn et al., 2012; Cho & Kim, 2010; Choi et al., 2011; Kim et al., 2010; Lee, 2011), other studies noted that adolescent and adult NKRs who stayed longer in third countries reported greater levels of psychological problems, including depression, PTSD, suicidal ideation, and alcohol abuse (Baek et al., 2007; Cho et al., 2011; Jung & Choi, 2017; Kim, 2010, 2013; Kim et al., 2011; Shin et al., 2016). Given that longer periods of time in third countries were related to an increased number of traumatic experiences among NKR (Kim et al., 2010), longer stay may thus increase the mental health risks posed by distressing or traumatic experiences in the immigration process (Baek et al., 2007).

Forced repatriation and the experience of failure to escape are also mental health risk factors for NKRs, as those refugee experiences are often accompanied by torture or threat of extermination (Cohen, 2012). About 20–32% of NKRs reported having attempted to escape more than once (Choi et al., 2009; Shin et al., 2016) and 26% had experienced at least one repatriation (Choi & Kim, 2011). NKRs who experienced forced repatriation consistently reported a greater degree of overall psychopathological symptoms, including PTSD and paranoid ideation (Choi & Kim, 2011; Jung & Choi, 2017; Kim et al., 2013; Shin & Kim, 2015), but not of somatization (Yoo, Lee, Koo, & Jun, 2017).

#### 3.2.2. Post-settlement factors

##### 3.2.2.1. Time since resettlement

The association between time since resettlement and psychological symptoms may differ between adolescent and adult populations of NKRs. For adolescent NKRs, a shorter duration of stay in South Korea was associated with higher prevalence or severity of both internalizing and externalizing problems in most studies (Baek et al., 2007; Choi et al., 2011; Lee, 2011; Shin & Kim, 2015), except for a few studies noting no significant association with depression (Cho et al., 2011; Kim, Park, & Park, 2014). Given the tendency of improvement in mental health with time observed in other young refugee populations (Montgomery, 2010), adolescent NKRs may experience more psychological and behavioural disturbances in the early years of settlement and adapt better with time, although the time effect may differ depending upon the context in resettlement locations (Reed, Fazel, Jones, Panter-Brick, & Stein, 2012).

On the other hand, for adult NKRs, most studies noted a lack of an association between time since resettlement and depression (Cho et al., 2005; Jeon et al., 2009; Kim et al., 2010; Nam et al., 2016; Shin et al., 2016; Song et al., 2016; Um, Chi, Kim, Palinkas, & Kim, 2015; Yoo et al., 2017), and two other studies even reported that female adult NKRs who had been resettled in South Korea for longer periods experienced greater depressive symptoms than did those in an earlier stage of settlement (Kim, 2012a; Lee, Chang, & Jun, 2016). Adult NKRs might have to deal with diverse challenges more directly, such as economic struggles, which might inhibit their psychosocial adaptation even after several years, whereas young NKRs might become more resilient under the protection of schools and parental support. However, these previous cross-sectional studies are not sufficient to conclude the long-term trajectory of psychological adaptation of young and adult NKRs.

##### 3.2.2.2. Acculturative stress

Along with traumatic experiences, resettlement stress in adjusting to a new culture is another important challenge for refugees (Berry, 1986). Particularly in societies with a single dominant culture such as South Korea, NKRs can experience greater cultural conflicts and stress. Acculturative stress includes homesickness, a sense of alienation, culture shock, feeling marginalized, and perceived discrimination (Kim et al., 2015). The adverse psychological impact of acculturative stress was consistently noted in prior findings, including decreasing NKRs’ self-efficacy and resiliency and increasing passive coping strategies for stressors (Kim et al., 2015; M. Lee et al., 2016; Lim & Han, 2016). Consequently, a high level of acculturative stress was found to predict adverse psychiatric outcomes such as depression, anxiety, and PTSD (Cho et al., 2011, 2009; Y. Kim, 2013; Kim et al., 2015; Lee, 2011; M. Lee et al., 2016; Lim & Han, 2016; Um et al., 2015), even after controlling for traumatic experience or daily life stress (Jeon et al., 2013). Furthermore, acculturative stress was suggested to mediate the adverse effects of risk factors, such as traumatic experience or perceived discrimination, on psychiatric outcomes (Lee, 2011; M. Lee et al., 2016). These findings highlight the crucial role of cultural adaptation in NKRs’ mental health.

##### 3.2.2.3. Social support

Particularly for NKR youths, social support seems to have a profound influence on psychological adaptation. Higher perceived social support consistently predicted better psychological adaptation (Baek et al., 2007; Kim et al., 2014; Park & Yoon, 2007), with higher unofficial social support – such as support from family members and friends – being particularly strongly related to lower depression and anxiety symptoms (Park & Yoon, 2007). Interestingly, when separating friends into other NKRs and South Koreans, only friendships with South Korean peers, but not with those from North Korea, were related to fewer internalizing problems in adolescent NKRs (Baek et al., 2007). This may be because building social connections with South Korean friends facilitates NKRs’ socio-cultural adaptation to South Korea.

However, the effect of perceived social support was mixed for adult NKRs. Whereas social support was linked to decreased psychiatric symptoms such as depression in two reports (Lim, Shin, & Kim, 2010; Song et al., 2016), after controlling for socio-demographic variables and familial factors, perceived social support had no significant association with depressive symptoms (Jeon et al., 2009). The independent mental health effect of social support for adult NKRs remains unclear, given the scarcity of findings.

##### 3.2.2.4. Family relationships

The association between family composition in South Korea and NKRs’ mental health is mixed in existing reports. In several reports, young and adult NKRs who had escaped from North Korea with their family members or living with family in South Korea presented lower levels of psychiatric problems, such as depression, anxiety, and PTSD symptoms (Ahn et al., 2012; Cho & Kim, 2010; Cho et al., 2011, 2009; Jeon et al., 2009; Lee, 2011). However, other studies found no significant association of psychiatric symptoms with being accompanied by family when escaping to South Korea (Kim et al., 2010) or the number of family members living together (Choi et al., 2011; Kim et al., 2014; Nam et al., 2016). Alternatively, some studies have shown that adult NKRs who were accompanied by their family and children to South Korea were more likely to experience depression and somatic symptoms compared to those who were not accompanied by family members (Kim, 2010). This might be due to economic pressures, which would increase with the number of family members, or the adverse effect of family conflicts on their psychological adaptation.

The effect of marital status on mental health has also shown some variation. Married NKRs were found to have a lower prevalence of suicidal ideation and higher levels of depressive symptoms than never married, separated, or divorced NKRs in several findings (Choi & Kim, 2011; Shin et al., 2016; Song et al., 2016). However, in other reports, being married and co-habiting with a spouse had no significant relationship with outcomes such as depression, anxiety, or PTSD symptoms (Cho et al., 2009; Jeon et al., 2009). Specifically, in one report (Cho et al., 2009), only marriage in North Korea was linked with increased depressive symptoms, while marital status in South Korea did not predict the mental health status of NKRs.

Unlike other family relationship factors, qualitative indexes of family functioning, such as family adaptability and emotional closeness, have consistently predicted NKRs’ decreased psychological problems, including depression (Nam et al., 2016; Um et al., 2015). Additionally, children of NKRs and adolescent NKRs presented less depressive and PTSD symptoms when having greater family support and less conflicts (Shin & Kim, 2015), and feeling greater attachment with parents (Emery et al., 2015). This implies that the qualitative characteristics of family relationships, rather than their quantitative aspect, have a more consistently significant impact on NKRs’ psychological adaptation.

##### 3.2.2.5. Socio-economic status (SES)

Low SES may put NKRs, especially adults, at a greater mental health risk. Adult NKRs with lower monthly income – especially those with family incomes under US$1000 – were more vulnerable to a wide array of psychiatric symptoms, including depression, anxiety, and somatization, in most reports (Ahn et al., 2012; Jeon et al., 2009; Kim, Lee et al., 2011; Nam et al., 2016; Shin & Lee, 2015; Song et al., 2016), but not all (Cho et al., 2009; Yoo et al., 2017). In particular, no independent relation was noted between low income and depressive symptoms when controlling for other significant variables, such as sociocultural adaptation, family characteristics, and physical health (Cho & Kim, 2010; Um et al., 2015). This suggests that NKRs’ financial resources might influence psychiatric symptoms through variables such as family relationship or health conditions. However, there is a lack of research examining the effect of SES or family income on NKR youths, except for one study noting no significant association with depression (Emery et al., 2015).

#### 3.2.3. Personal factors

##### 3.2.3.1. Individual characteristics

As suggested in the stress–diathesis model (Monroe & Simons, 1991), individual differences in NKRs might influence their ability to cope with environmental stressors greatly, thus affecting mental health outcomes. For instance, resilience was closely associated with NKRs’ psychological adaptation (Jung, Son, & Lee, 2013; Y. Kim, 2013; Kim et al., 2015; Lee, 2011; Nam et al., 2016). Other studies further revealed that resilience has a mediating role in the association of psychiatric problems with a variety of risk and protective factors. Specifically, acculturative stress was linked to increased depression and anxiety symptoms through decreased ego resiliency in one report (Kim et al., 2015); in another report, NKRs’ resilience mediated the link between family cohesion and attenuated depressive symptoms (Nam et al., 2016). These consistent results suggest a critical role of NKRs’ resilience in coping with stress, including social adaptation difficulties.

Additionally, individual coping strategies against internal and external stressors are linked to mental health outcomes. Among psychological defence mechanisms, which refer to automatic psychological coping processes (American Psychiatric Association, 1994; Cramer, 2000), the tendency to use more adaptive coping strategies (e.g. sublimation and humour) and conversely, less maladaptive coping strategies (e.g. passive-aggressive behaviour, regression, dissociation) were linked to fewer psychological problems, such as decreased anxiety, interpersonal sensitivity, and hostility (Jun et al., 2015; Kim, 2010). NKRs’ psychiatric symptoms were also predicted by passive coping style to avoid problems (Lim et al., 2010; Lim & Han, 2016). In another report, NKRs having more difficulty in emotion identification and expression – alexithymia – were found to have a greater negative impact of traumatic experience on PTSD symptoms, suggesting the significant role of adaptive emotional coping in attenuating the aftermath of stressful events (Park et al., 2015).

##### 3.2.3.2. Sex

Empirical findings on sex differences in NKRs’ mental health problems are mixed. In more than a half of studies, no sex differences were found in NKRs’ psychiatric outcomes (Choi et al., 2011; Emery et al., 2015; Jeon et al., 2009; Kim, 2012b; Y. Kim, 2013, 2016; Lee, 2011; Nam et al., 2016; Park et al., 2015; Um et al., 2015); however, the remainder of studies noted consistent patterns of sex differences in various psychological problems. Specifically, female NKRs appear to be more vulnerable to experience emotional and internalizing problems, including depression, anxiety, suicidal ideation, and somatization (Cho et al., 2009; Jun et al., 2015; Kim et al., 2011; Kim & Shin, 2010; 2015; Kim et al., 2008; S. Kim et al., 2011; Y. Kim et al., 2010; Shin et al., 2016). The prevalence of PTSD was also found to be higher among females (Kim et al., 2010). In contrast, male NKRs tend to present more behavioural and externalizing problems, including alcohol problems (Baek et al., 2007; Cho et al., 2011; Jeon et al., 2008; Kim & Shin, 2010; 2015).

##### 3.2.3.3. Age

Studies have shown different results for the association of age with psychiatric symptoms in young and adult NKRs. These results must be interpreted with caution, given the difficulties in differentiating aging, period, and cohort effects as well as other confounding factors (Fazel et al., 2012). For NKR youths, four studies noted no significant relationship between age and mental problems such as depression, anxiety, and PTSD (Cho et al., 2011; Choi et al., 2011; Emery et al., 2015; Y. Kim, 2013), while the remaining two studies showed more internalizing problems and interpersonal difficulties among older youths than younger ones (Baek et al., 2007; Kim & Shin, 2015). It is possible that NKR youths have a greater likelihood of experiencing psychological difficulties at older ages during adolescence.

For adult NKRs, most studies suggested a greater risk for overall psychopathology among older NKRs, including PTSD, depression, substance use, and somatization (Chang & Son, 2014; Choi & Kim, 2011; Choi et al., 2009; Jun et al., 2015; Kim, 2010; Kim & Shin, 2010; Kim et al., 2013; M. Lee et al., 2016; Lim et al., 2010; Park et al., 2015; Yoo et al., 2017), albeit not all studies (Cho & Kim, 2010; Cho et al., 2009; Kim et al., 2010; Song et al., 2016; Um et al., 2015). Particularly, compared to those in their early adulthood (20s to 30s), older adults (40s to 50s) seemed to be more vulnerable to mental health problems (Choi & Kim, 2011; Kim & Shin, 2010).

The generally higher levels of mental health problems among older adults are perhaps due to their higher exposure to traumatic events in North Korea or during their defection, as older adult NKRs were found to experience more traumatic events in one report (Park et al., 2015). Other explanations for the age differences in psychiatric symptoms may include aging effects on mental health, older NKRs’ cohort characteristics, age-related policies, and the longitudinal effects of traumatic events.

##### 3.2.3.4. Physical health condition

In general, physical health condition appears to have a close association with NKRs’ mental health problems. Specifically, self-reported general physical health was negatively linked to poor psychological adaptation, including higher levels of anxiety, depression, and PTSD symptoms in young and adult NKRs (Ahn et al., 2012; Baek et al., 2007; Cho et al., 2011; Y. Kim, 2013). In adult NKRs, the number of physical illnesses and having at least one chronic health complaint were also predictive of overall psychological problems, including depression and anxiety (Cho & Kim, 2010; Cho et al., 2009; S. Kim et al., 2011; H. Kim et al., 2011).

## 4. Discussion

Consistent with other refugee studies indicating poorer mental health outcomes of refugees in comparison with non-refugees (Fazel et al., 2005; Porter & Haslam, 2005), young and adult NKRs appear to have a higher prevalence or level of psychiatric symptoms, particularly PTSD and depression. Table 3 presents the consistently identified risk and protective factors of their mental health.

**Table 3.** Summary of risk and protective factors.

Variables	Domain	Direction	Number of studies (author, year)	Total no. of subjects
Traumatic experience	Pre-settlement factors	Risk	11 (Baek et al., 2007; Cho & Kim, 2010; Cho et al., 2011; Y. Choi et al., 2017; Emery et al., 2015; W. Jeon et al., 2013; H. Kim, 2012b; Y. Kim, 2013, 2016; Y. Kim et al., 2015; Y. Lee et al., 2016; S. Lim & Han, 2016; J. Park et al., 2015)	2747
Longer stay periods in third country	Pre-settlement factors	Risk	7 (Baek et al., 2007; Cho et al., 2011; Y. Jung & Choi, 2017; H. Kim, 2010, 2013; H. Kim et al., 2011; H. Shin et al., 2016)	2131
Educational level in North Korea	Pre-settlement factors	Protective	5 (Cho et al., 2011; Y. Choi et al., 2009; H. Kim, 2010; Y. Kim et al., 2010; D. Song et al., 2016)	1405
Forced repatriation	Pre-settlement factors	Risk	3 (B. Choi & Kim, 2011; Y. Jung & Choi, 2017; S. Kim et al., 2013; H. Shin & Kim, 2015)	2959
Acculturative stress	Post-settlement factors	Risk	7 (Cho et al., 2011; Cho et al., 2009; W. Jeon et al., 2013; Y. Kim, 2013; Y. Kim et al., 2015; K. Lee, 2011; M. Lee et al., 2016; S. Lim & Han, 2016; Um et al., 2015)	1421
Low income*	Post-settlement factors	Risk	6 (Ahn et al., 2012; B. Jeon et al., 2009; H. Kim et al., 2011; Nam et al., 2016; G. Shin & Lee, 2015; D. Song et al., 2016)	1000
Social support	Post-settlement factors	Protective	5 (Baek et al., 2007; L. Kim et al., 2014; J. Lim et al., 2010; Y. Park & Yoon, 2007; D. Song et al., 2016)	955
Family relationship quality	Post-settlement factors	Protective	4 (Emery et al., 2015; Nam et al., 2016; H. Shin & Kim, 2015; Um et al., 2015)	982
Older age*	Personal factors	Risk	10 (Chang & Son, 2014; B. Choi & Kim, 2011; Y. Choi et al., 2009; Jun et al., 2015; H. Kim, 2010; H. Kim & Shin, 2010; S. Kim et al., 2013; M. Lee et al., 2016; J. Lim et al., 2010; J. Park et al., 2015; Yoo et al., 2017)	4169
Female sex (for emotional problems)	Personal factors	Risk	8 (Cho et al., 2009; Jun et al., 2015; H. Kim et al., 2011; H. Kim & Shin, 2010, 2015; J. Kim et al., 2008; S. Kim et al., 2011; Y. Kim et al., 2010; H. Shin et al., 2016)	2270
Poor physical health	Personal factors	Risk	7 (Ahn et al., 2012; Baek et al., 2007; Cho & Kim, 2010; Cho et al., 2011; Cho et al., 2009; H. Kim et al., 2011; S. Kim et al., 2011; Y. Kim, 2013).	1302
Male sex (for behavioural problems)	Personal factors	Risk	5 (Baek et al., 2007; Cho et al., 2011; W. Jeon et al., 2008; H. Kim & Shin, 2010, Kim & Shin, 2015)	1187
Resilience	Personal factors	Protective	4 (J. Jung et al., 2013; Y. Kim, 2013; Y. Kim et al., 2015; K. Lee, 2011; Nam et al., 2016)	874

*Indicates that the whole study sample comprised adult NKRs.

When multiple studies used the same sample, we counted that sample once.

For NKRs, there are distinct factors that contribute to adverse mental health states: exposure to traumatic events in their home country and during migration, and resettlement stress related to sociocultural adaptation. In line with prior research on other refugee and forcibly displaced populations (Eisenbruch, 1991; Lustig et al., 2004; Porter & Haslam, 2005), traumatic experience and acculturative stress were of the most frequently studied and consistently identified mental health risks for NKRs. In addition, the immigration process involving longer stay periods in third country and the experience of repatriation can aggravate their mental health risks. These stressors, therefore, represent unique external challenges to NKRs’ psychosocial adaptation. Based on these results, we strongly encourage tailored psychological interventions aimed at reducing the adverse consequences of traumatic experiences, as well as social interventions for creating a receptive atmosphere to ultimately alleviate acculturative stress and facilitate socio-cultural adaptation.

Positive social and familial relationships are effective buffers against effects of adverse life events on mental health. Social support is a critical protective social factor, especially for the psychological adaptation of young NKRs. As found in a refugee study by Gorst-Unsworth and Goldenberg (1998), affective support from personal relationships (e.g. family, friendships) plays a more pivotal protective role than does official support in NKRs’ mental health (Park & Yoon, 2007). In particular, friendships with South Korean peers were predictive of better adaptation among adolescent NKRs (Baek et al., 2007). However, adolescent NKRs have a lack of opportunities to interact with South Korean peers and might experience difficulty in building social connections with them (Keum, Kwon, & Lee, 2004). Efforts should be made to increase opportunities for NKRs to interact with South Korean peers both at school and community levels. Additionally, aiding NKRs to overcome their communication difficulties (Baek et al., 2007) would help them to develop connections with South Koreans. Regarding familial factors, the qualitative characteristics of family relationships were consistently associated with NKRs’ positive mental health outcomes. Interventions aiming to improve the family bonds of NKRs and preventing the family-related stressors, such as economic overburden or family conflicts, are needed to enhance NKRs’ resilience and ability to adapt to the new society (Nam et al., 2016).

Personal factors such as individual characteristics and socio-demographic factors further explain NKRs’ individual differences in psychological adaptation. Among individual characteristics, self-reported resilience has been found to be a consistent protective factor of the mental health of young and adult NKRs. Given the critical role of individual coping capability in moderating the psychological consequences of refugee status (Hooberman, Rosenfeld, Rasmussen, & Keller, 2010), guidance on the use of productive and adaptive coping strategies, such as active coping and emotion regulation, would be effective for NKRs’ psychological adaptation. Poor physical health or chronic illness among NKRs was found to be associated with mental vulnerability. Thus, integrated care targeting both physical and mental health might be effective for NKRs. The sex differences in the occurrence of different psychiatric symptoms among NKRs are consistent with previous findings on refugees’ mental health (Fazel et al., 2012): female sex was found to be a risk factor for emotional problems, such as depression and anxiety, while male sex was a risk for behavioural problems, such as substance abuse. We also identified older age as a robust risk factor for mental health problems, especially among adult NKRs. Additionally, considering that adult NKRs with low income or low educational levels might be at a greater risk of mental problems, economic and educational support is encouraged.

However, the aforementioned findings are derived mainly from cross-sectional studies that cannot identify causal relationships between risk and protective factors and psychiatric outcomes. Therefore, longitudinal research is needed to determine long-term causality between variables and also to understand the pathways underlying the psychological influences of such factors. Furthermore, future research from a longitudinal perspective will help to clarify the long-term trajectory of NKRs’ psycho-social adaptation after resettlement.

Despite the extensive need for mental health interventions for NKRs, most South Korean government interventions seem incapable of meeting these individuals’ specific psychological needs (Kang & Lee, 2009). Although all NKRs are provided with a 12-week compulsory education that seeks to improve their social adaptation before their settlement in South Korea, this programme tends to focus more on political and economic education and job training (Chung & Park, 2014). After resettlement in South Korea, the scope of government support includes residence, employment, and health insurance, all of which are essential for their social adaptation; nevertheless, these forms of support cannot fully meet their mental health needs. Importantly, we found only a few intervention studies specifically designed for NKRs (e.g. Kang, Lee, & Lee, 2010). In order to provide tailored interventions for improving NKRs’ mental health, more scientific research on the design and evaluation of psychological interventions is needed.

We noted several limitations of this review study. Firstly, due to the heterogeneity of studies concerning clinical outcomes, instruments, and study populations (e.g. different age groups), we could not use meta-analytic techniques to synthesize the data and summarize findings. Secondly, relevant articles published in languages other than English or Korean (e.g. Chinese) may have been omitted. Thirdly, publication bias should be considered; as we only included published articles, there may be a risk of inflation of results.

## 5. Conclusions

Overall, as the first review study of the mental health of NKRs, this research highlights that they are a mentally vulnerable population requiring extensive clinical attention. To better predict and prevent adverse mental health outcomes, rigorous studies that investigate the long-term interactions between NKRs’ mental health outcomes and pre- and post-settlement environmental factors as well as personal characteristics are needed. Furthermore, future studies should aim to develop and implement psychological interventions targeting this specific population through adequate policies.

## Highlights

- We reviewed 56 empirical studies on mental health of North Korean refugees (NKRs) in South Korea
- NKRs had a high prevalence and severity of psychiatric symptoms, including depression, anxiety, and PTSD symptoms.
- We identified consistent risk/protective factors for NKRs’ mental health problems
- The comparability of the studies was limited by their heterogeneity.
